# Prevalence of urinary cotinine levels in children under 5 years of age during consultations for acute respiratory disease at the emergency department of the Universidad de La Sabana clinic

**DOI:** 10.1186/s12887-020-02193-8

**Published:** 2020-06-16

**Authors:** María Fernanda Tovar, Wendy Ortiz, María Alejandra Valderrama, Fabio Rodríguez, Oscar Gamboa, María José Maldonado, Sergio Iván Agudelo

**Affiliations:** 1 School of Medicine, Universidad de La Sabana, Chía, Colombia; 2 Universidad de La Sabana, Chía, Colombia; 3 Universidad de la Sabana Clinic, School of Medicine, Universidad de La Sabana, Chía, Colombia

**Keywords:** Smoke, Nicotine, Cotinine, Tobacco products, Tobacco smoke, Air pollutants, Preschool, Respiratory disorders, Passive smoking, Second-hand smoke

## Abstract

**Background:**

Several environmental factors favour the occurrence of acute respiratory disease, which is the main reason for paediatric consultations in our country (Colombia). Tobacco smoke is considered a significant environmental pollutant with a great impact on health. The objective of this study is to estimate the prevalence of cotinine levels measured in urine, in children between 1 to 60 months of age who attended an emergency department with acute respiratory disease.

**Methods:**

A cross-sectional study was conducted that included children between 1 and 60 months of age with acute respiratory disease who were admitted to the emergency department of the Universidad de La Sabana Clinic between April and July 2016.

**Results:**

We included 268 patients and 36% were female. Of the total population examined, 33.96% showed positive results for urinary cotinine, of whom 97.8% had values between 10 and 100 ng/ml, which is considered positive for exposure to second-hand smoke. The principal pathology was recurrent wheezing in 43.96% of these cases. Regarding the presence of smokers at home, it is important to mention that in 54.95% of the children with positive urinary cotinine test was no related with smokers at home. And in 45.05% of positive urinary cotinine was evidence of smokers at home, being associated with the positive result *P* <  0.001 and smoking within the house *P* = 0.018; smoking when children were present did not have significant *P* = 0.105. The activities performed after smoking such as hand washing, change of clothes, eating, brushing teeth, did not influence the test result *P* = 0.627.

**Conclusions:**

A high prevalence of urinary cotinine was observed, which is associated with the presence of a smoker at home, and this relationship was independent of the activities performed by the smoker after smoking. In addition, a positive test for urinary cotinine was presented in some children without documented exposure to cigarette smoke inside the home, which may be explained by the presence of environmental cotinine. Therefore, it is necessary to perform educational interventions aimed at parents and caregivers who smoke.

## Background

Acute respiratory disease (ARD) in children represents a problem of epidemiological relevance due to its elevated dissemination potential and the associated high prevalence of morbidity and mortality [1]. Additionally, it generates high social and economic costs, representing 40 to 60% of paediatric consultations in developing countries such as Colombia [2]. Some environmental factors favour the presentation of ARD, such as intra and extra-domiciliary environmental pollution, and may increase its incidence [1].

Tobacco smoke is considered an important environmental pollutant [3]. The current prevalence of cigarette consumption in Colombian adults between 18 and 69 years of age is 12.8%, and the rate is higher in men. Frequent inhalation can lead to the generation of different pathologies, mainly involving the respiratory and cardiovascular systems [4]. In addition, tobacco smoke remains the leading cause of preventable death worldwide (6 million people per year) [5]. This toxic and invisible mixture is composed of gases and particles, including carcinogens and heavy metals such as arsenic, lead, and cyanide. The residue clings to walls and ceilings and is absorbed into carpets, clothing, curtains, upholstery, vehicle interiors, and other items [6, 7].

Three forms of contamination exist: first-, second-, and third-hand smoke. First-hand smoke refers to smoke that is inhaled and exhaled directly while smoking, and the person mainly affected is the smoker. Second-hand smoke occurs secondary to combustion when the cigarette is lit, and third-hand or residual smoke is the one that remains on surfaces and in dust, interacts with other compounds, and may persist for several hours or even days after the cigarette is smoked [6].

According to the Global Youth Tobacco Survey, approximately 50% of the children in the world are exposed to second-hand smoke [8]. Children are more vulnerable to complications secondary to that exposure because they breathe faster than adults, which allows them to aspirate more harmful chemicals per kilogram of weight than an adult in the same time period [9].

Cotinine is the main metabolite of nicotine and an important biomarker of exposure to second-hand smoke that can be found in the blood, saliva, and urine. This metabolite has a half-life of approximately 15–17 h; therefore, it is considered the best biochemical marker of second-hand smoke [10]. Elevated levels of urinary cotinine are most commonly associated with second-hand smoke exposure [11].

### Aim and objectives

Because of the potential harm to children that can be caused by exposure to cigarette smoke and to measure the magnitude of the problem, considering the absence of local data, the main objective of this study was to estimate the prevalence of cotinine levels measured in the urine of children between 1 month and 5 years old with ARD who were admitted to the emergency department of the Universidad de La Sabana Clinic.

## Methods

### Type of study and population

A cross-sectional study was conducted including children between 1 and 60 months of age who were admitted to the emergency department of the Universidad de La Sabana Clinic between the months of April and July 2016. In a previous research [12], we did an endemic channel for respiratory diseases in our population and geographic location, that allowed us identify the months with highest disease prevalence and to get a representative sample for this study. The inclusion criteria were the following: diagnosis of acute respiratory infection (ICD-10 J00X-J22X) that required observation or hospitalization. Informed consent was signed by the parents. Patients with pre-existing genitourinary disease pathology (renal insufficiency, glomerulopathies, hydronephrosis) or with a current diagnosis of urinary tract infection, patients with chronic cardiac or pulmonary disease under paediatric subspecialty management, and those with parental dissent were excluded.

The NicAlert® test was used to measure urine cotinine levels, and this test has a sensitivity of 95% and a specificity of 97% [13]. This test, designed for the semi-quantitative determination of urinary cotinine levels, determines whether an individual has been exposed to tobacco products during the prior 48 h. The reference values are as follows: level 0: cotinine concentration between 1 and 10 ng/ml; level 1: between 10 and 30 ng/ml; level 2: between 30 and 100 ng/ml; level 3 between 100 and 200 ng/ml; level 4: between 200 and 500 ng/ml; level 5: between 500 and 1000 mg/ml, and level: 6 greater than 1000 ng/ml. Levels 1 to 3 were considered to be positive for exposure to second-hand smoke [13]. A single sample was taken per participant.

Data related to the demographic and clinical characteristics of the patients were collected using an electronic instrument previously designed to capture this information. The severity of respiratory disease was classified using the Wood-Downes scale [14]. The clinical information was obtained from the electronic history of each patient.

### Sample size

The sample size was estimated using the normal approximation with the following parameters: prevalence 50%, type I error 5% (two-tailed), distance between the population proportions 6%. Using these parameters, a sample size of 267 subjects was estimated.

### Statistical analysis

Descriptive analyses were performed using measures of central tendency (median) and dispersion (range) for continuous variables. Absolute and relative frequencies were used for categorical variables. The overall period prevalence with its 95% confidence interval was estimated.

We explored the variables of exposure at home that are related to the presence of urinary cotinine. Additionally, the association of these variables with the presence of complications was determined. Regarding the exploratory objectives, for continuous variables, Student’s t-test was used to analyse independent samples, and the assumption of normality was verified using the Shapiro-Wilk test. If this assumption was not met, the nonparametric Wilcoxon rank sum test was used. For the categorical variables, contingency tables were constructed, and independence tests were performed using the Chi-square test or Fisher’s exact test. Two-tailed analyses were conducted with a type 1 error level of 5%. The STATA 11® program was used for the analysis.

## Results

**Fig. 1:**
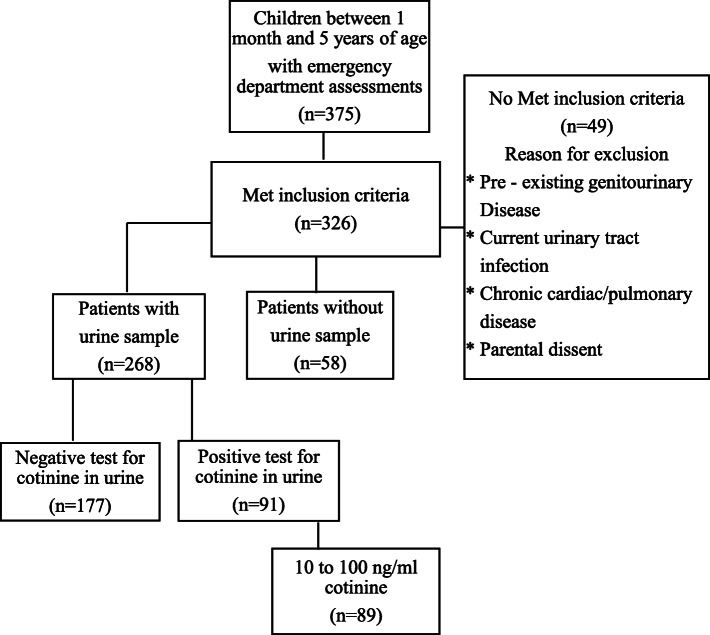
Study population

The Universidad de La Sabana Clinic covers the municipalities of the Central Sabana Province (*Sabana Centro*) of the Department of Cundinamarca in Colombia. During the months of April to July 2016, 326 patients met the inclusion criteria, among whom a urine sample was no able to obtain in 58 patients, resulting in a total of 268 patients included in the analysis, the Fig. 1 show the population selection process for this study.

The age range of the children who participated in the study was between 1 and 60 months, with a median of 12 months, and 36% were girls. A similar distribution of urban (59.62%) and rural residents (40.38%) was observed and no statistically significant difference was observed (*p* > 0.05). The other demographic and clinical characteristics of the patients are shown in Table 1.

**Table 1:** Sociodemographic and clinical characteristics

Characteristic		
**Sex**	**n**	**%**
Male	169	63.06
Female	99	36.94
**Age (median)**	**Months**	**Range**
	12	1–60
**Location of residence**	**n**	**%**
Urban area	158	59.62
Rural area	107	40.38
**Diagnosis**	**n**	**%**
Bronchiolitis	95	35.45
Croup	5	1.87
Pneumonia	77	28.73
Recurrent wheezing	91	33.96
**Complications**	**n**	**%**
Yes	75	27.99
No	193	72.01
**Smokers in the Home**	**n**	**%**
Yes	70	26.12
No	198	73.88

Of the total population studied, 33.96% had a positive test for cotinine in the urine. Of these, 97.8% had levels between 10 and 100 ng/ml (see Table 2). The predominant pathologies were recurrent wheezing in 43.96% of the patients, and bronchiolitis and pneumonia, each one, in the 27.47% of the patients.

**Table 2:** Prevalence of cotinine levels in the urine

Cotinine prevalence		
	n	%
**Positive**	91	33.96
0–30 ng/ml	86	94.5
30–100 ng/ml	3	3.3
100–200 ng/ml	2	2.1
**Negative**	177	66.84

Regarding the presence of smokers at home, 26.12% of all children were exposed to cigarette smoke, as reported by the parents, interestingly, 45.05% of whose had positive cotinine test had smokers at home and in 56.1% of the cases, people to do this activity inside the home. Associations statistically significant were found between a positive test result and children who live with smokers *P* <  0001 and smoking within the home *P* = 0.018. Activities performed after smoking such as hand washing, change of clothes, eating, brushing teeth did not influence the test result (see Table 3).

**Table 3:** Cotinine levels according to characteristics related to smoke habits

	Cotinine Levels		
	Positive	Negative	*p* value
**Smokers in the house**			
Yes	41 (45.05)	29 (16.38)	< 0.001
No	50 (54.95)	148 (83.62)	
**Place of smoking**			
Inside the home	23 (56.1)	8 (27.59)	0.018
Outside the home	18 (43.9)	21 (72.4)	
**Smoking in the presence of the children**			
Yes	10 (24.39)	2 (6.9)	0.105
No	31 (75.61)	27 (93.1)	
**Activities after smoking**			
Yes^a^	25 (60.98)	16 (55.17)	0.627
No	16 (39.02)	13 (44.83)	

^a^ Hand washing, Change of clothes, Eating, Brushing teeth

Finally, into the general population, we observed some complications such as: use of oxygen at home, hospitalization in paediatric intensive care unit (ICU), presence of atelectasis and pleural effusion. But we should perform further studies to get more information about the association of complications and positive Urinary cotinine.

## Discussion

The present study is the first conducted in Colombia to objectively detect the urine cotinine levels and determine the relationship with the behaviour of caregivers and ARD. Exposure to cigarette smoke is one of the major risk factors for respiratory disease [15], which is one of the main causes of medical consultations for children under 5 years of age. It is therefore of great importance to demonstrate the negative impact of this practice on the health of children.

We found a high prevalence of urinary cotinine (33.96%) in children admitted to the emergency department for respiratory pathology, the result of the study are consisted with the results of a study by Wilson KM et al. [16], where a level of exposure to cigarette smoke of 40% was documented in children hospitalised with the influenza virus. These results should alert the health community to reinforce public health measures aimed at promoting healthy habits such as not exposing children to this environmental pollutant, which is important because significant circulation of the N1H1 influenza virus has been found in the months of highest rainfall in Colombia [17].

An extensive search was conducted, and only one similar study was identified, which was conducted by Jenny Pool et al. and titled Exposure of children to second hand smoke in England [18]. This study used the same method for cotinine detection and documented positive levels of between 10 and 100 ng/ml in 96% of the cases, similar to the level found in the present study (97.8%). Additionally, one participant had a positive cotinine level, but no smokers lived in the home. This result was associated with second-hand smoke in school, similar to the results of our study, in which 54.95% of the positive test was no related with smoker at home, which compels consideration of environmental pollution. Reports from the World Health Organization in 2010 showed the presence of environmental cotinine in some public places in Bogotá [19]. No similar Latin American studies were identified; therefore, this study provides relevant information.

Likewise, children with a recurrent wheezing diagnosis had a higher proportion of positive urine levels of cotinine. This finding is a possible indicator of exacerbation of the pathology due to exposure to this environmental pollutant, as described in different studies that state that children exposed to tobacco smoke have an increased risk of developing recurrent wheezing syndrome [20–22]. This risk is increased according to the number of cigarettes smoked inside the home. In a study conducted by Kalliola et al. [21], 43% of Finnish children who were exposed to tobacco smoke, according to their parents’ report, had deteriorated lung function.

A study conducted by the University of California in 2006 [23] demonstrated that the separation of smokers and non-smokers within the same space with shared air does not eliminate or minimise the exposure of non-smokers to second-hand smoke. This finding is supported by the results of the present study, which showed a higher number of children with a positive cotinine value when caregivers smoked inside the home [23]. It should be considered that actions such as smoking outside the home, washing hands, changing clothes, brushing teeth, or eating are factors that can reduce exposure to second-hand smoke. However, these actions are not entirely protective because cotinine remains in the environment, impregnated into surfaces, implying a non-perceived exposure for minors. This is contrary to the perception of parents, who consider that exposure is reduced by 100% when performing these cleaning actions. This finding is supported by a study by Jenny Pool et al. [18], in which no difference in exposure was observed between those who smoked outside or inside the home. Thus, education and awareness of parents and caregivers regarding this practice and its impact on the health of the child is very important. Therefore, during the data collection period, interventions for the parents and caregivers of patients with a positive test were conducted, stressing the importance of ending this habit that harms the health of their children.

In the present study, it was not possible to determine the association between the presence of cotinine in the urine and complications of respiratory pathology because cotinine was associated with a lower risk of these complications. However, in the study, only the age variable was assessed as a risk factor for complications, without considering other individual factors. Therefore, the findings could not be verified because an adjusted measure could not be obtained. These findings should be verified in subsequent studies. Furthermore, because the NicAlert® test detects exposure during the prior 48 h, the presence of cotinine in the urine cannot be excluded for children who have smoking parents when the test is conducted beyond 48 h and a negative result is obtained, despite the existence of an epidemiological nexus reported by caregivers.

This study presented some limitations; due to the functioning health system, the follow-up was difficult because the participants came from different regions and post hospitalization controls would not be carried out into of the Univerisidad de la Sabana Clinic, for this reason was impossible established the morbidity into the populations studied.

## Conclusions

Despite the fact that our findings suggest that most of the positive urinary cotinine test were related to the presence of smoker at home, as well as the development of this activity in the presence of the children, it is important to mention that the most relevant finding of our study was to evidence patients with positive urinary cotinine tests without documenting exposure to cigarette smoke inside the home, that may be explained by the presence of environmental cotinine. Further studies are required to assess environmental cotinine as well as the association of complications in respiratory patients. Assessing urinary cotinine levels can be used as a non-invasive marker for exposure to cigarette smoke and is a practical test method for children.

Furthermore, there is a misconception among parents and smokers that some activities after smoking, such as hand washing, change of clothes, eating, brushing teeth, can be protective Against second-hand exposure in children. Taking into account the above, it is considered necessary to carry out educational interventions aimed at smoking parents and caregivers.

## Data Availability

If you require more information about all data obtained during this study please contact to corresponding author.

## Abbreviations

ICD: International Classification of Diseases
ARD: acute respiratory disease
